# Differences in electrode placements between consensual and nonconsensual electroconvulsive therapy: retrospective chart review study

**DOI:** 10.1192/bjo.2025.10053

**Published:** 2025-06-25

**Authors:** Hye-Sang Shin, Naveen Thomas, Yiting Amanda Gong, Rajeev Krishnadas, Alby Elias

**Affiliations:** Division of Mental Health and Wellbeing, Western Health, Melbourne, Australia; Faculty of Medicine, Dentistry and Health Science, University of Melbourne, Melbourne, Australia; Department of Psychiatry, University of Cambridge, Cambridge, UK; Department of Psychiatry, University of Melbourne, Melbourne, Australia

**Keywords:** Electroconvulsive therapy, electrode placement, ECT, Mental Health Act, consent

## Abstract

**Background:**

Electroconvulsive therapy (ECT) is often used to treat severe mental disorders in individuals with impaired capacity to consent to the treatment. Little is known about how different types of electrode placement are used in consensual and nonconsensual ECT.

**Aims:**

To investigate whether there was an association between ECT consent status and electrode placement, given that ECT electrode placement affects efficacy and cognitive outcomes.

**Method:**

Using a statewide database across 3 years in Victoria, Australia, we performed chi-squared tests to determine whether consent status (consensual versus nonconsensual) was associated with particular electrode placements. A three-way log–linear analysis was then conducted to examine whether age, gender, level of education and psychiatric diagnosis influenced the relationship between consent status and electrode placement. Given the comparable cognitive outcomes of right unilateral and bifrontal ECT, these electrode placements were combined in the analysis.

**Results:**

In total, 3882 participants received ECT in the Victorian public health service during the study period. In the nonconsensual ECT group, 722 of 1576 individuals (45.81%) received bitemporal ECT, compared with 555 of 2306 (24.06%) in the consensual group (*χ* ^2^ = 200.53; *P* < 0.0001; odds ratio: 2.6673, 95% CI: 2.3244–3.0608). This association remained significant after adjustment for gender, age, level of education and diagnosis.

**Conclusion:**

Significantly more participants in the nonconsensual ECT group received bitemporal ECT rather than right unilateral or bifrontal ECT compared with those in the consensual group. As bitemporal ECT is associated with more cognitive impairment, this choice of electrode placement in vulnerable patients who lack capacity to consent raises ethical considerations in the practice of ECT.

Electroconvulsive therapy (ECT) is an effective treatment option for various psychiatric disorders. ECT is a highly regulated treatment in many jurisdictions worldwide. In the Australian state of Victoria, where the present study was conducted, patients must ordinarily provide consent to treatment with ECT. However, as ECT is often reserved for individuals with more severe psychiatric conditions, patients’ capacity to consent to treatment may be impaired. Severity of symptoms is one factor that predicts impaired capacity.^1^

When this study was conducted, ECT in Victoria was regulated by provisions in division 5 of the Mental Health Act 2014^2^ (this was replaced by the Mental Health and Wellbeing Act 2022, which is essentially the same as the Mental Health Act 2014 with respect to the administration of ECT). According to the Mental Health Act 2014, ECT may be performed on a patient who is not a young person if: (a) the patient has personally given informed consent in writing to the performance of a course of ECT on himself or herself; or (b) the tribunal has granted an application for the performance of a course of ECT made under section 93. Under section 93, an authorised psychiatrist may make an application to the tribunal to perform a course of ECT on a patient who is not a young person if the patient does not have capacity to give informed consent to the performance of a course of ECT on himself or herself. When ECT was administered under a tribunal order, it was known as nonconsensual ECT. However, the fine aspects of treatment, such as electrode placement and dosing, were decided by the treating team in consultation with patients and families after consideration of effectiveness and side-effects.

In terms of its delivery, ECT has undergone several advances during the years since its inception, leading to the emergence of various electrode placements and stimulus pulse widths tailored to suit clinical indications and patient preferences.^3,4^ The commonly used electrode placements are bitemporal, right unilateral and bifrontal. Bitemporal ECT has a long history and is believed to provide superior efficacy in severe conditions such as catatonia; however, recent literature has challenged this view, particularly concerning depressive disorders.^5–7^ Although some data suggest that bitemporal ECT may be associated with faster remission, there is no significant difference in eventual remission rates between patients treated with bitemporal ECT and those receiving high-dose right unilateral ECT.^8,9^ By contrast, right unilateral and bifrontal ECT are associated with fewer cognitive deficits than bitemporal ECT.^9–11^ For instance, there is evidence to suggest that bitemporal ECT is more often associated with autobiographical memory deficits that persist for up to 6 months after a course of ECT.^11^ However, bitemporal ECT is still commonly used and is often preferred in the treatment of patients with the most severe forms of psychiatric disorders, such as catatonia and delirious mania, where rapid improvement is critical, despite emerging evidence of greater side-effects and no difference in eventual remission rates.^8^

Given that patients treated with nonconsensual ECT are more likely to have a more severe capacity-impairing illness, they may disproportionately receive bitemporal ECT compared with other electrode placements. For example, Finnegan et al explored the relationship between illness severity and consensual versus nonconsensual ECT administration.^12^ They found that the proportion of extremely ill patients was significantly higher in the nonconsensual ECT group than in the consensual group. Notably, the proportions of patients receiving bitemporal ECT were relatively high in both groups [46 (96%) *v*. 79 (83%)]. There was little information about comparison with other electrode placements. These findings indicate a possible practice bias skewed towards the use of bitemporal ECT. Thus, there is a need for careful consideration of electrode placement while administering ECT, especially with those who do not have the capacity to consent to treatment and/or limited ability to express this choice.

In the present study, we tested the hypothesis that patients treated with nonconsensual ECT may disproportionately receive bitemporal ECT in comparison with other electrode placements across statewide services in Victoria, Australia.

## Method

### Study design and participants

This study was a retrospective review; hence, it did not involve interventions or interactions with individual research participants. The Department of Health Human Research Ethics Committee approved the study ([reference: HREC/73840/DOH-2021-291014(v3)]. The study investigated the types of electrode placement used in Victorian public mental health services for patients who received ECT during 2017 and 2020. The data were obtained from the Victorian Agency for Health Information. We excluded data from 2020 to 2021 owing to the impact of COVID-19 on healthcare services. The study included only adult participants above 18 years of age to avoid concerns about identification and proxy consent for minors. The public health data included age range, gender, education level, diagnostic indication for ECT and electrode placement. Pulse width information about brief and ultra-brief pulses was not available. Participants who received more than one type of electrode placement during the same episode of treatment were not included in the study. As the data were collected over years, participants may have had multiple ECT episodes in one calendar year before and after their date of birth, resulting in duplication in entries for age stratified data (16 patients).

The study only used summary data, including stratified age ranges and education levels; individual raw data were not available for analysis. This ensured data anonymity. According to the guidelines set out by the National Statement on Ethical Conduct in Human Research 2023, the local Human Research Ethics Committee granted a waiver of consent according to section 2.3.9 of the above document, on the basis that only group-level summary data were accessed and utilised in this study, and individual data were not accessed. This ensured adequate protection of the privacy of participants.

### Analysis

We performed chi-squared tests to examine whether consent status (consensual versus nonconsensual ECT) was associated with electrode placement (bitemporal versus unilateral versus bifrontal). To examine whether age, gender, education status or diagnosis influenced the relationship between consent status and electrode placement, we performed a three-way log–linear analysis. For example, to determine whether the association between consent status and electrode placement was affected by age, we examined whether there was a three-way interaction among consent status, electrode placement and age (consent status × electrode placement × age).

## Results

Overall, there was a statistically significant association between consent status (nonconsensual versus consensual) and electrode placement (bitemporal versus both bifrontal and unilateral; *χ* ^2^ = 91.56 and *χ* ^2^ = 184.1, respectively, *P* < 0.0001 for both). We then conducted additional chi-squared tests to determine where the significant associations were. The results of this initial analysis are shown in Table 1.

**Table 1:** Between-group differences in electrode placement for those who received nonconsensual versus consensual ECT under the Mental Health Act

Consent status	Bitemporal	Bifrontal	Unilateral	Total	Bitemporal versus bifrontal	Bitemporal versus unilateral	Bifrontal versus unilateral
Nonconsensual ECT	722	252	602	1576	*χ* ^2^ = 91.56, *P* < 0.001	*χ* ^2^ = 184.19, *P* < 0.001	*χ* ^2^ = 1.18, *P* = 0.27
Consensual ECT	555	481	1270	2306	Odds ratio 2.48 (95% CI: 2.05–2.99)	Odds ratio 2.74 (95% CI: 2.36–3.17)	Odds ratio 1.10 (95% CI: 0.92–1.32)

With respect to bitemporal versus bifrontal placement, 722 (72.14%) patients treated with nonconsensual ECT received bitemporal treatment compared with 555 (53.57%) patients who had consensual ECT. This difference was statistically significant (*χ* ^2^ = 91.56, *P* < 0.001; odds ratio = 2.48; 95% CI: 2.05–2.99). Similarly, with respect to bitemporal versus unilateral placement, 722 (54.53 %) patients in the nonconsensual ECT group received bitemporal treatment compared with 555 (30.41%) patients who underwent consensual ECT. This difference was also statistically significant (*χ* ^2^ = 184.19, *P* < 0.001; odds ratio = 2.74; 95% CI: 2.36–3.17). However, in the analysis of bifrontal versus unilateral placement, 252 (29.50%) patients who had nonconsensual ECT were no more likely to receive bifrontal than right unilateral treatment, compared with 481 (27.47%) patients in the consensual ECT group (*χ* ^2^ = 1.08; *P* = 0.29; odds ratio = 1.10; 95% CI: 0.92–1.32).

Given that there was no significant difference in consent status between bifrontal and unilateral placement in our data, we combined the numbers of people who received unilateral and bifrontal electrode placements. A further analysis comparing bitemporal with combined bifrontal and unilateral placement showed that 22 (45.81%) patients treated with nonconsensual ECT received a bitemporal treatment, compared with 555 (24.06%) patients in the consensual ECT group. This difference was statistically significant (*χ* ^2^ = 200.53; *P* < 0.0001; odds ratio = 2.66; 95% CI: 2.3244–3.0608). In other words, patients treated with nonconsensual ECT were 2.66 times more likely to receive bitemporal treatment instead of other placements than patients treated with consensual ECT.

Next, we tested whether age, gender, education status and diagnosis influenced the above relationship. The results of this analysis are shown in Table 2. Among those above 65 years old, 159 (33.75%) patients who had nonconsensual ECT received a bitemporal treatment, compared with 194 (23.33%) patients treated with consensual ECT. This difference was statistically significant (*G* ^2^ = 16.21; *P* < 0.001). Similarly, among those below 65 years old, 563 (50.9%) patients in the nonconsensual ECT group had a bitemporal placement, compared with 361 (24.47 %) in the consensual ECT group. This difference was also statistically significant (*G* ^2^ = 192.98; *P* < 0.001). This suggests that nonconsensual ECT was more likely to involve bitemporal placement than consensual ECT, independent of age group. Among females, 355 (42.77%) patients who had nonconsensual ECT group received a bitemporal treatment, compared with 326 (23.69%) patients who had consensual ECT (statistically significant at *G* ^2^ = 86.94; *P* < 0.001). Among males, 367 (48.03%) patients who had nonconsensual ECT had a bitemporal placement, compared with 229 patients (24.65 %) in the consensual ECT group (statistically significant at *G* ^2^ = 109.17; *P* < 0.001). This suggests that nonconsensual ECT was more likely to involve bitemporal placement, independent of gender. Among those with more than 11 years of education, 433 (45.96%) patients treated with nonconsensual ECT had a bitemporal placement, compared with 344 (23.61%) patients treated with consensual ECT. This was statistically significant at G^2^ = 129.22; *p* < 0.001. Among those with education of<11 years, 289 (45.58%) patients who had nonconsensual ECT received a bitemporal treatment compared to 211 (24.85%) patients in the consensual ECT group (statistically significant at *G* ^2^ = 69.61; *P* < 0.001), suggesting that nonconsensual ECT was more likely to involve a bitemporal placement, independent of education status. Among depressed patients, 239 (37.81%) patients in the nonconsensual ECT group had a bitemporal placement, compared with 384 (21.31%) patients in the consensual ECT group (statistically significant at *G* ^2^ = 63.63; *P* < 0.001); and among patients with other diagnoses, 483 (52.55%) patients who had nonconsensual ECT received a bitemporal placement, compared with 171 (35.11%) patients in the consensual ECT group (statistically significant at *G* ^2^ = 37.99; *P* < 0.001). These findings suggest that nonconsensual ECT was more likely to be a bitemporal treatment, independent of diagnosis status. Briefly, our results suggest that patients treated with nonconsensual ECT were more likely to receive bitemporal ECT irrespective of age, gender, education status and diagnosis.

**Table 2:** Between-group differences in electrode placement for those receiving consensual versus nonconsensual ECT grouped according to age, education status, sex and diagnosis

	Consensual ECT		Nonconsensual ECT		Statistic^a^	*P*-value
Demographic and diagnosis	Bitemporal, *n* (%)	Unilateral + bifrontal, *n* (%)	Bitemporal, *n* (%)	Unilateral + bifrontal, *n* (%)		
Age						
>65 years	194 (23.33%)	637 (76.65%)	159 (33.75%)	312 (66.24)	*G* ^2^ = 16.21	<0.001
<65 years	361 (24.47%)	1114 (75.52%)	563 (50.95%)	542 (49.04)	*G* ^2^ = 192.98	<0.001
Number of years in education						
>11	344 (23.61)	1113 (76.38)	433 (45.966)	509 (54.03)	*G* ^2^ = 129.22	<0.001
<11	211 (24.85)	638 (75.14)	289 (45.58)	345 (54.42)	*G* ^2^ = 69.61	<0.001
Gender						
Female	326 (23.69)	1050 (76.30)	355 (42.77)	475 (57.22)	*G* ^2^ = 86.94	<0.001
Male	229 (24.65)	700 (75.34)	367 (49.19)	379 (50.80)	*G* ^2^ = 109.17	<0.001
Diagnosis						
Depression	384 (21.30)	1418 (78.69)	239 (37.81)	393 (62.18)	*G* ^2^ = 63.63	<0.001
Other	171 (35.11)	316 (64.88)	483 (52.21)	442 (47.78)	*G* ^2^ = 37.99	<0.001
Total	555	1751	722	854	*χ*2 = 200.53	<0.0001

a. *G* ^2^ is equivalent to *χ* ^2^ in a log–linear analysis.

## Discussion

The present study compared the frequencies of different electrode placements in patients who underwent consensual and nonconsensual ECT. We found that individuals who were treated with nonconsensual ECT were more likely to undergo bitemporal electrode placement compared with right unilateral or bifrontal placement, in contrast to those treated with consensual ECT. This is consistent with previous research that reported a significantly higher proportion of bitemporal ECT in patients who had nonconsensual ECT.^12^ In addition to replicating this finding in a larger sample, our study also identified comparative differences in electrode placements across various diagnostic indications, age groups, gender and educational status. These differences were evident irrespective of the patient’s diagnostic status. In other words, the differences were not explained by nondepressive indications such as catatonia, delirious mania and psychotic disorders, which may have been overrepresented in the nonconsensual ECT group and for which bitemporal ECT has traditionally been selected.^5,8^ For patients in public health settings with severe or urgent conditions, bitemporal ECT may be considered a favourable and rational choice.^8^ However, in the present study, we did not have data on severity and response to treatment, making it difficult to determine whether illness severity specifically moderated electrode placement and response to ECT.

Our findings further emphasise the importance of considering the cognitive side-effects associated with bitemporal ECT, especially for patients undergoing nonconsensual ECT who may lack the capacity to consent to treatment and comprehend this risk. Although ECT itself does not exclude patient participation in discussions about various aspects of treatment, including electrode placement, the severity of illness may hinder patients’ ability to express choices. Previous research has indicated inadequate communication of cognitive deficits between practitioners and patients, with memory problems being reported as one of the most distressing side-effects of ECT.^13,14^

Our study had several strengths and limitations. The strengths include its being the first to compare frequencies across electrode placements in relation to consent status, focusing on clinical practice data collected from a large sample size across a state-wide population and thereby increasing the generalisability of the findings to public settings. The study was further bolstered by its use of relatively objective variables, such as electrode placements and consent status, as opposed to self-reported symptoms that may be susceptible to recollection and reporting biases. However, drawing conclusions on clinical efficacy from these data should be approached with caution, as the study lacked efficacy data and indicators of cognitive side-effect burden rates across these populations, both at baseline and during treatment.

It is essential to note that our findings do not imply the superiority of one electrode placement over others. Our study was a cross-sectional examination of associations and, as such, does not provide insight into the potential causes of the observed discrepancy. Therefore, no definitive causal conclusions can be drawn from our study. To gain a deeper understanding, a longitudinal prospective study that explores individual-level data, as well as data from clinicians detailing the rationale behind their choice of a particular electrode placement over another during individual decision-making, would be necessary.

In conclusion, our findings have important implications for patient participation in choosing electrode placement based on the risk/benefit ratio, even in the case of nonconsensual ECT, considering the increased cognitive impairment associated with bitemporal ECT. These findings underscore the importance of advocating for the safety and well-being of a highly vulnerable group of patients during ECT.

## Data Availability

Data are available upon request from the corresponding author, R.K.
